# Navigating complexity to support justice-involved youth with FASD and other neurodevelopmental disabilities: needs and challenges of a regional workforce

**DOI:** 10.1186/s40352-021-00132-y

**Published:** 2021-02-27

**Authors:** Rebecca Anne Pedruzzi, Olivia Hamilton, Helena H. A. Hodgson, Elizabeth Connor, Elvira Johnson, James Fitzpatrick

**Affiliations:** 1 Telethon Kids Institute, PO Box 855, West Perth, Western Australia 6872 Australia; 2 Mercy Services, 32 Union St, Newcastle, NSW 2297 Australia; 3 School of Psychological Science, The University of Western Australia, Perth, 6009 Australia

**Keywords:** Justice, Youth, Fetal alcohol spectrum disorder, FASD, Neurodevelopmental disabilities, Workforce development

## Abstract

**Background:**

Young people with Fetal Alcohol Spectrum Disorder (FASD) can face significant challenges in their lives, including overrepresentation in the justice system from a young age. Police questioning and court proceedings can be difficult for these young people to navigate. Practice and policy responses are necessary to identify these individuals, provide appropriate support/rehabilitation, and upskill the justice workforce. The aim of this research was to determine the unmet workforce development needs of a regional workforce providing care and support to youth involved with the justice system. Interviews were conducted with 29 participants from 14 organisations to understand the support provided to youth, existence and uptake of referral pathways, and unmet needs.

**Results:**

Results revealed a workforce that wants to see improvements to outcomes for young people with FASD and other neurodevelopmental disabilities who enter the youth justice system. However more support is required through training, ongoing funding, and assistance to develop FASD informed work practices.

**Conclusions:**

The workforce supporting youth to navigate the justice system requires practical interventions to achieve best practice so that young people with FASD and other neurodevelopmental disabilities receive the support that they need. Following the interviews a model of care tool was developed and piloted in the sector. The tool includes current pathways through the justice system and provides resources to assist staff in achieving best practice care for young people with FASD and other neurodevelopmental disabilities.

## Background

The criminalisation of young people with complex physical and mental health needs is widespread (McCausland & Baldry, 2017). Data from Australian jurisdictions demonstrates that many young people in detention present with a neurodevelopmental disorder, mental illness, drug and alcohol use, and/or histories of trauma and victimisation (Australian Institute of Health and Welfare, 2018; Bickel & Campbell, 2002; Bower et al., 2018; Frize, Kenny, & Lennings, 2008; Haysom, Indig, Moore, & Gaskin, 2014; Perry & Newbigin, 2017). Other groups are also disproportionately affected by the current justice system. Indigenous Australians are incarcerated at nearly eighteen times the rate of the non-Indigenous population (Weatherburn, 2014) and the rate of disparity has worsened since the 1991 Royal Commission into Aboriginal Deaths in Custody, increasing at different rates across the states and territories of Australia (Weatherburn & Holmes, 2017). The overrepresentation of both Indigenous Australians and people living with disabilities in the justice system demonstrates the effects of ongoing systemic disadvantage (Baldry, McCausland, Dowse, McEntyre, & MacGillivray, 2016; Dowse, Baldry, & Snoyman, 2009; Smith & Dowse, 2019). This systemic disadvantage has also been noted internationally (Burd, Fast, Conry, & Williams, 2010), (McLachlan, 2017; Mclachlan et al., 2019; Momino et al., 2012; Popova, Lange, Bekmuradov, Mihic, & Rehm, 2011).

In Australia, Fetal Alcohol Spectrum Disorder (FASD) has been identified as being of specific concern in the youth justice system (Australian Institute of Health and Welfare, 2018). FASD is a disorder caused by prenatal alcohol exposure that includes neurodevelopmental problems in at least three domains (Motor skills; Cognition; Language; Academic Achievement; Memory; Attention; Executive Function, including impulse control and hyperactivity; Affect Regulation; and Adaptive Behaviour, Social Skills or Social Communication) (Bower et al., 2017). Secondary issues associated with living with FASD may include problems with the law, inappropriate sexual behaviour, vulnerability to substance abuse and mental health problems (Douglas, Hammill, Hall, & Russell, 2012; Douglas, Hammill, Russell, & Hall, 2012; Streissguth et al., 2004). These effects impact significantly upon individuals, families and communities, through increased rates of chronic disease, increased caregiver stress, incarceration, and loss of productivity (Popova, Lange, Burd, & Rehm, 2015; Reid, Akison, Hoy, & Moritz, 2019; Reid, Moritz, & Akison, 2019). The occurrence of FASD across Australia is difficult to estimate due to limited diagnostic capacity, under-reporting of cases (Burns, Breen, Bower, O'Leary, & Elliott, 2013), the complexity of diagnosing the disorder generally (Roozen et al., 2016) and a possible hesitancy of women to report alcohol consumption during pregnancy due to stigma. However, studies in specific populations have found rates of FASD amongst the highest in the world (Bower et al., 2018; Fitzpatrick et al., 2017). A recent study by Bower et al. (2018) conducted in the only youth detention centre in Western Australia found 89% of youth in the centre had at least one domain of severe neurodevelopmental impairment. Furthermore, 36% of these youth were diagnosed with Fetal Alcohol Spectrum Disorder (FASD).

Individuals living with FASD typically face several systemic and social disadvantages that can increase their involvement with the justice system. Individuals with FASD can be highly suggestible, have difficulty with impulse control and connecting actions with consequences. Police questioning and court proceedings are especially problematic as individuals may aim to please the interviewer rather than providing factual information (Fast & Conry, 2009). Further, they may experience difficulties with expressive and receptive communication, and therefore struggle to express themselves or comprehend court proceedings (Kippin et al., 2018; Snow & Powell, 2008). The behaviours of young people living with FASD may be negatively judged by both the justice system and broader society (public stigma), and individuals with FASD may have low self-esteem (self-stigma) (Corrigan et al., 2019; Corrigan & Kosyluk, 2014). Families and caregivers of young people with FASD and other neurodevelopmental delays may experience self-stigma and feelings of failure when the young people in their care are criminalised because of their disability (McCausland & Baldry, 2017).

For youth with FASD involved in the justice system, the recognition of their condition is crucial to procedural justice (McLachlan, Mullally, Ritter, Mela, & Pei, 2020) and the application of effective care and support strategies (Hamilton et al., 2019; McLachlan et al., 2020). Case law reviews suggest if FASD is recognised and impacts upon culpability, sentencing outcomes may be more appropriate. Importantly adaptations can be made to programs, treatments, and discharge plans if the system supports such an approach. However, numerous barriers impact the capacity of the custodial and non-custodial workforce to provide appropriate support and care for these youth. These barriers occur across several socioecological levels and include a lack of specific FASD knowledge (Masotti et al., 2015; McLachlan et al., 2020; Passmore et al., 2018), insufficient training to recognise and manage youth with FASD and neurodevelopmental impairments generally, (Ellem & Richards, 2018; Mogavero, 2016; Passmore et al., 2018), inadequate information sharing processes to support young people (Appleby, Shepherd, & Staniforth, 2019; Hamilton et al., 2019; Passmore et al., 2018), and insufficient provision of resources for staff and youth (Hamilton et al., 2019; McLachlan et al., 2020).

Practice and policy responses are necessary to improve access to FASD diagnosis, provide appropriate support and rehabilitation, and upskill the justice workforce in the appropriate management of individuals with FASD (Bower et al., 2018). The national FASD strategy action plan in Australia has prioritised work in the criminal justice system, to ‘provide education and training for staff in juvenile justice systems and community policing, including identification processes and referral pathways for further assessment and support’ (Commonwealth of Australia Department of Health, 2018, p. 32). The plan also prioritises diagnosis for youth in correctional settings, and the development of better models of management, support and care including non-custodial therapeutic options that enable courts to ‘divert offenders identified with neurodevelopmental or cognitive impairments, including FASD, away from prisons and into programs and services’.

To gain maximum impact from this plan, training and care models must be based on the best available evidence, link to strategic plans and initiatives, and be developed in collaboration with key stakeholders both in the workforce and the community (Agency for Clinical Innovation, 2013; Appleby et al., 2019; Fitzpatrick et al., 2020; Tremblay et al., 2017). International research has long called for FASD and other neurodevelopmental disabilities training for justice professionals (Mutch, Watkins, Jones, & Bower, 2013; Snoyman, 2010), yet evidence for such initiatives remains scarce ((Passmore et al., 2016; Reid, Kippin, Passmore, & Finlay-Jones, 2020). In the first study of its kind, Passmore et al. (2018, 2020) developed and evaluated a customised training intervention to enhance the capacity of custodial officers to respond to and manage FASD and similar neurodevelopmental disabilities in a youth detention setting. A pre-post design was employed to assess changes in knowledge and attitudes of this workforce. Results demonstrated significant positive shifts in workforce knowledge and attitudes post intervention. This research demonstrates the critical importance of developing a locally specific understanding of needs to create effective interventions in this setting.

To improve outcomes for young people with FASD and other neurodevelopmental disabilities, the current study assessed the specific needs of services providing support to justice involved youth in a regional Australian hub.

This paper reports on the results of workforce interviews which later resulted in a pilot of a Model of Care tool for this workforce.

## Methods

The research was conducted in Newcastle, a large coastal city of New South Wales, Australia, in the context of a national network of FASD prevention activities. Research was guided by a community reference group (Local Drug Action Team [LDAT]) comprising community, medical, education and legal expertise. Governance was provided through a research and FASD prevention collaboration. Ethics approval for this research was obtained from the University of Western Australia Human Research Ethics Committee (#RA/4/20/5032).

### Participants

**Fig. 1:**
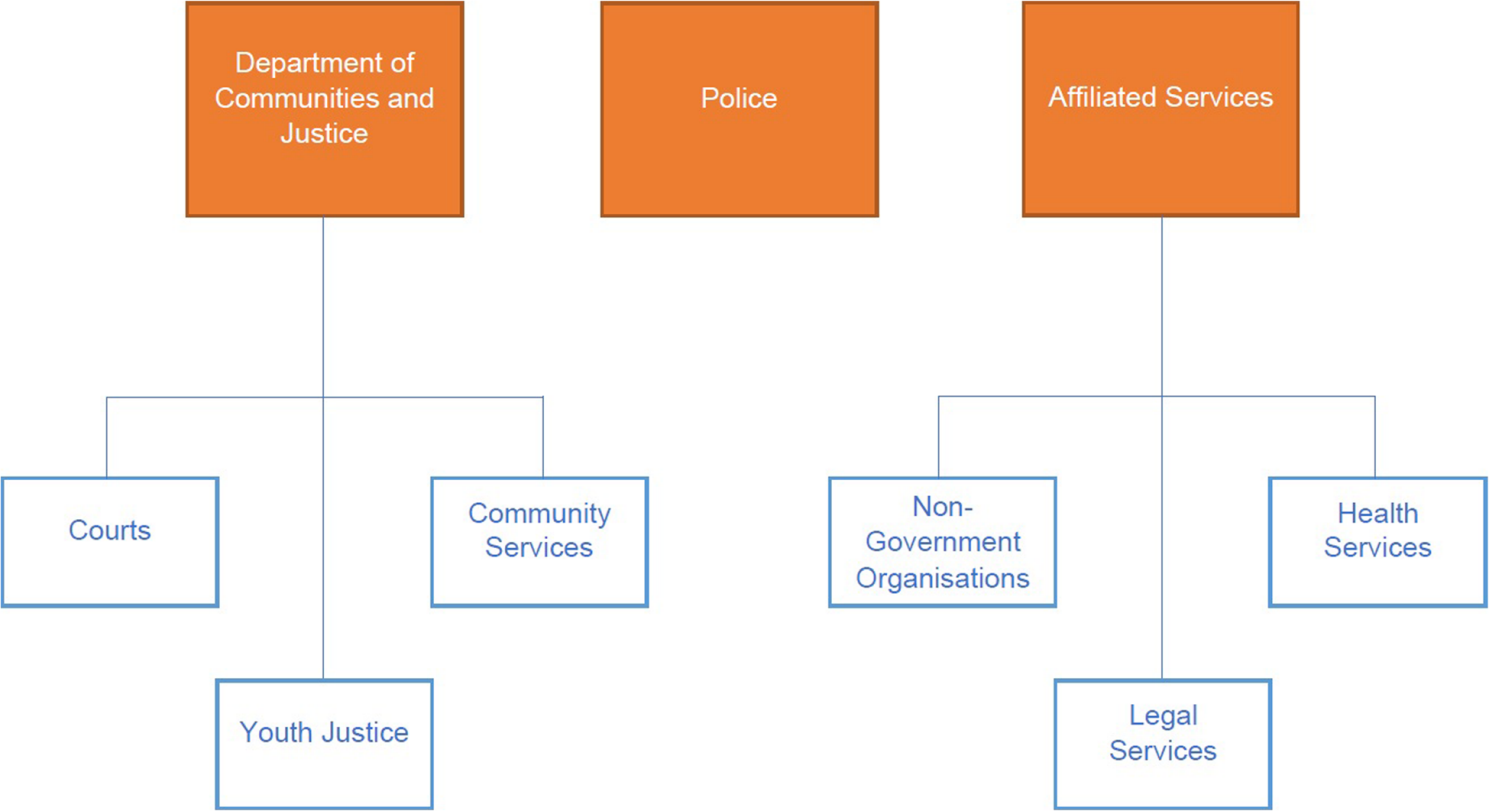
Youth justice services in the study area

Participants were staff members employed by numerous services in the greater Newcastle area. These included police, courts, legal, health, employment and welfare, and affiliated non-government organisations including disability support (see Fig. 1). All staff were invited to participate via email and a follow-up phone call. Initially, 32 organisations were contacted. Snowball sampling was then used to contact staff working at a further 4 organisations. Interviews were conducted with 29 staff from 14 organisations / government departments. Roles of participants are presented in Table 1. There were no incentives offered for participation.

**Table 1:** Workforce roles of participants

Role	Frequency
Case manager / case worker	7
Clinical staff	6
Police	2
Legal professional	6
Mediation or advocacy	5
Program manager	3
**Total**	29

### Measures / instruments

Semi -structured interview questions were developed with the research project team, the LDAT, and a steering committee. Refinement of this process was undertaken by piloting the questions with partner organisations involved in the research. The primary focus of the research was FASD, however flexibility was key to the interview process given overlap between FASD and other neurodevelopmental disabilities, and issues of missed diagnosis and misdiagnosis (Petrenko, Tahir, Mahoney, & Chin, 2014).

### Procedure

A desktop review, two community forums, and an environmental scan were conducted to identify programs and services (both government and non-government) that support young people in contact with, or at risk of contact with, the justice system in Newcastle (Hodgson et al., 2020). Government reports provided a primary source of information (Association of Children’s Welfare Agencies, 2016; Justice Health & Forensic Mental Health Network and Juvenile Justice NSW, 2017; Murphy, McGinness, Balmaks, McDermott, & Corriea, 2010) and a list of key organisations and individuals were compiled from this review. Further information was identified via the LDAT and the local FASD co-ordinator employed on the project. Snowball sampling was used to contact additional organisations in the area. Participants were contacted by phone or face-to-face to gain consent for individual interviews, which were conducted by the team, audio-recorded, and transcribed by a professional transcription service.

### Data management and analysis

Qualitative data collected from the interviews was audio-recorded and stored on a password-protected network. Interviews were analysed using thematic analysis. Descriptive codes were developed from a first read-through of the interview transcripts. The codes were classified under two main groupings: codes relating to clients/young people; and codes relating to service delivery. The codes identified under the ‘clients/young people’ group included: diagnosis, stigma, trauma, criminogenic risk, young person’s understanding, rights of young person and victimisation. The codes identified under the ‘service delivery’ group included: staff knowledge/skills, criminal responsibility, welfare needs, system requirements, communication with client, service gaps, referral pathways and staff burnout. Transcripts were coded in an excel spreadsheet. Following further discussions and a review by all members of the research team, the codes were refined and key themes developed. Thematic analysis was conducted using Excel version 12.1.

## Results

Results are presented under the four key themes of: diagnosis and outcomes, complex needs, complex roles, and navigating the system. These themes fit within a social-ecological model, moving between factors that impact the individual (in this case, both the young person and the service worker), through relationships (the young person’s family/home life/trauma histories) and social structures (the role of services in providing support to young people), to social norms (the expectations that society has around safety, the role of the justice system, and stigma, amongst others) and policy (for example, the legislation that governs assessments of criminal responsibility).

### Theme 1: diagnosis and outcomes

Participants across youth justice, the courts, and affiliated services identified several issues related to diagnosing FASD in the local area. Some staff suspected FASD in young people who had not received a diagnosis or had been diagnosed with other conditions such as attention deficit hyperactivity disorder (ADHD) or autism spectrum disorder (ASD). This was especially so when family histories of alcohol misuse were noted.

*They may have had something that was like an ASD or oppositional or whatever kind of diagnosis. But I actually think when you hear their history, and definitely their parents' history or their mum's history, it's very likely that they probably also have FASD. (Source: Interview #14)*

Participants discussed both a lack of diagnosis and diagnostic pathways, with many families and young people unable to afford both the cost of travel and the waiting period to access diagnostic services further afield.

*I know they diagnose in Western Australia, and I know that it can be done in Sydney, but it's very expensive. I don't know local services that are doing the diagnosis. (Source: Interview #5)*

The process of referring young people elsewhere for diagnosis was described by one participant as a lengthy and complex process.

The other thing that I've done is try to get people down to ***** to have a full battery of assessments down there. I think one of the challenges is that when a child starts to display difficulties, what assessments do you undertake to begin with? Where do people go? Do they go to OTs, do they go to [speech therapists], do they go to [psychologists], do they look at autism, do they look at ADHD, do they look at behavioural charts, do they look at intellectual disability? ... There are so many facets there … and many of them coming from challenging backgrounds in terms of developmental trauma. That's another thing that we also need to recognise. (Source: Interview #7)

In some instances, the lack of diagnosis was explained by a reluctance to follow through with medical assessment on the part of both the young person and the staff member, because of the stigma associated with a FASD diagnosis, while other participants suggested that diagnosis did not occur because young people had disengaged from services, and diagnosis was not perceived as beneficial.

*They don't understand what a diagnosis is going to help them achieve, so there's no push on it. Their families, most likely, probably, see that the same way. There's so much crisis going on that that's the last thing that they're thinking about. (Source: Interview #12)*

Another issue raised several times throughout the interviews was the need to understand the purpose of a diagnosis. Participants wanted to know how, if at all, a FASD diagnosis would benefit their clients, questioning whether it would change the way that they work with clients, and whether a diagnosis would enable access to greater support, for example through the National Disability Insurance Scheme (NDIS).

*What I've learnt about it is the treatment's going to be exactly the same. So, diagnosed with global development delay or intermittent explosive disorder or whatever, they're going to receive exactly the same treatment as they would for FASD. The only difference is the NDIS funding now, so you - if the treatment has such limited positive outcomes in any event, the treatment is the same regardless of whether you get a diagnosis. (Source: Interview #13)*

*But where [a diagnosis of FASD] takes you from there, I honestly don't know, because … in terms of extra support for the child, I don't know how - where that takes me, a FASD diagnosis. (Source: Interview #29)*

In contrast, some participants spoke strongly in favour of diagnosis, arguing that it would bring benefits such as more appropriate interventions and recognition of mitigating factors before the law.

*Where we determine that a young person doesn't … have capacity to understand the legal proceedings, we'll request a report from a psychologist or psychiatrist … it may be that we look at a Section 32, which is where the court puts a treatment plan in place and deals with them under mental health legislation rather than proceeding with the matter at law. So, we do have different options available to deal with the matter before the court, depending on what those issues are. (Source: Interview #28)*

All participants wanted to know how to best assist the young people they worked with. Diagnosis of FASD was perceived as one way to do so, when it was clearly linked to improved outcomes for the young person. However, participants were reluctant to push for a diagnosis of FASD when it was perceived as a redundant activity.

### Theme 2: complex needs

Participants recognised that the young people entering the justice system had faced a series of adverse events in their lives, including trauma, family breakdown, drug and alcohol issues, illiteracy, disengagement from services, and social isolation.

*There aren’t many kids that I deal with in juvenile justice or in my role in police … that have mum dad and kids that are functional. Everybody loving. It just doesn’t happen like that. A lot are living with grandparents, aunties, out of home [care]. (Source: Interview #1)*

In some instances, participants described young people who had become entrenched in the system from a very young age, moving through multiple out-of-home care placements or finding themselves in placements where the carer was unable to provide adequate support. In other instances, young people were described as becoming entrenched in the system because detention provided benefits such as stability and shelter.

*A fair few of the young people that I've worked with that have kind of been from out-of-home care, a lot of time they tend to want to reoffend so that they can go back, get locked back up again because it's safer for them to be in than to be out. (Source: Interview #9)*

According to participants, young people with some form of neurodevelopmental disability often struggled to understand the court process, or to link cause and effect; this put them at perhaps greater risk of ongoing engagement with the justice system, due to repeated offending or failure to meet bail conditions and other obligations. Participants also reported needing to advocate for their clients, to ensure that they were not stigmatised or labelled as non-compliant, and that they were treated fairly. Participants described stepping in to provide linkages to welfare, medical and counselling services, to address the broader social, economic, and health needs of young people entering the justice system.

*Our main focus is around the criminogenic need and what might be contributing to their criminal behaviour and trying to reduce that. But we also try to address any welfare issues, get them in touch with the right services that could help out with employment, education, those sorts of things. We'll certainly give information around what sort of counselling services might be able to assist. (Source: Interview #4)*

The complexities faced by young people in the justice system meant that staff were at risk of burnout, particularly when interventions to assist young people were not successful or when service gaps meant that their clients’ needs could not be met.

*I think that everyone is trying so that there are incredibly good children's lawyers and that there are some very good children's magistrates and prosecutors. Everyone's being sympathetic and careful in the terminology that they use and pragmatic about how can we get to some solutions, but I'm sure that they are stressed about particular things and they're tuned off to some things and they are not feeling particularly engaged. (Source: Interview #13)*

Participants across the sector recognised the complexities faced by young people encountering the justice system, with FASD just one amongst many factors impacting on their journey. The need to balance the young person’s needs with the needs of the community and the requirements of the system increased stress levels amongst a highly passionate group of people who wanted to see the best possible outcomes for young people.

### Theme 3: complex roles

Participants described a range of different processes to achieve the best outcomes for the young people they worked with. In some instances, participants were involved in pilot projects that had been designed to address identified needs within the system. These participants were positive about the impact of their service or model, however they also expressed uncertainty about the future in a climate of competitive funding. One participant who began their working life overseas stated that understanding of FASD was, by comparison, lower in Australia. Other participants explained that much of their knowledge had been gained on the job whilst working elsewhere, in regional and remote areas of Australia.

While most people interviewed demonstrated at least some knowledge of the ways that FASD can present, there was also a strong call for training that would improve skills in working with young people with FASD. Participants perceived skills improvement as a way to improve outcomes for young people, and to help address the burnout experienced by some staff members when they felt their clients were slipping through the cracks.

*The first word that came to mind is the frustration, and that sounds really bad, but I feel like there's this frustration amongst some of the higher powers to say, these kids keep getting in trouble. They keep reoffending. Why? What does it mean? But they haven't really asked the right questions. (Source: Interview #12)*

*The issue is identifying it, primarily. There's just not really - no one seems to be using assessment tools to identify FASD and then having the appropriate information. (Source: Interview #28)*

Participants also reported that young people were often labelled as non-compliant or were not being provided with the supports they need to navigate the system.

*All the services around her closed their doors, they burn out from her. [They think] she's naughty and she's spoilt. But she doesn't have capacity. Even the family kind of go, oh, she's just a naughty teenager. Because that gives them an excuse not to provide her with the supports that she needs as a kid with potentially FASD. So, these are the issues. (Source: Interview #5)*

*Well they get labelled as non-compliant or that no interventions work, or they're kind of too hard basket, which then leads them to just going straight back to custody. (Source: Interview #15)*

Other participants recognised the need for early intervention, to prevent young people from becoming involved with the justice system in the first place.

*They are entitled to three cautions where they come in to the caution with a support person to speak to the youth liaison officer and they talk about the offence and what they can do … That’s where the alarm bells should be sounding … [And] police as another filter and it’s more important to pick it up before it comes to juvenile justice. They … never come straight to juvenile justice, the police are always the first step. (Source: Interview #1)*

Participants recognised that their own roles existed within a broader system of service delivery, and that outcomes for young people were dependent on the required services working together. Referral pathways and communication between services did not always work smoothly, with services tending to cluster in silos. Participants wanted to know where to direct young people, to ensure that their needs were being met. To a large extent, referral pathways relied on interpersonal relationships, which was perceived both positively and negatively. Networking across services was described as possible because of the smaller population compared to metropolitan regions. However, there were not always enough resources to address the existing need.

*We've got a hub of services and supports that we hope to grow. Being there - there's communication between the services that are there around who's working with what young person or supports they've already got in place. That's [great], compared to other courts where there's no supports at all. I think there's still a long way to go. There's no - it's similar to the mental health supports. There are not enough services for the amount of people who actually need help. (Source: interview #19)*

Similarly, participants who were time-poor or in roles that required extensive knowledge and skills struggled to develop adequate networks to respond to individual needs.

*Our problem is that we will have people present from all sorts of walks of life and then its where to direct them to. Some sort of directory or something like that would be awesome. To then say even a central point and go righty-o this person has this certain disorder if we leave you their details are you then able to point them in the right direction and then that happens that would be great. (Source: Interview #25)*

A common theme running throughout the interviews indicated that service workers were doing the best they could with limited resources. Participants identified regional variations in availability of services, with more services available in the city environment compared to nearby regional areas. Participants also identified issues with delayed access to services, resulting in a lack of options for diversion from the criminal justice system.

The complexities of the system and of their roles within it presented challenges for staff, who were passionate about seeking the best outcomes for young people. Strong interpersonal relationships with other staff across the region enabled extensive referrals; however these were often ad-hoc and dependent on the knowledge and experience of staff. Participants expressed frustrations with short term funding, regional variation in service availability, and difficulties navigating bureaucratic structures. However, they also demonstrated commitment to addressing the extensive needs of their clients, in many cases describing instances when they have gone over and above the requirements of their roles.

### Theme 4: navigating the system

A significant factor impacting on participants was the legal framework within which disabilities are recognised under law. The Mental Health (Forensic Provisions) Act 1990, commonly referred to in participant interviews as ‘Section 32’, details how magistrates can address issues of both mental illness and intellectual disability. The process of working within this legal framework affects how neurodevelopmental disabilities are perceived, as expressed by one participant who described the process of assessing someone’s cognitive ability at the point of first contact with police.

*We tend to broadly speak about things like mental illness. We are not trained as such to be able to distinguish. We rely very heavily upon the person who we are talking to divulging their own mental health history … In many cases it can be lost if the cognitive disability is subtle enough to a degree that it’s coming across as confusion or an inability to understand consequences. Or even being able to calm down behaviour, that sort of thing. In many cases a lot of things can be misinterpreted as something that it’s not. (Source: Interview #25)*

At the same time, those with responsibility for making legal decisions emphasised the role they had in weighing up knowledge of disabilities or impairments with knowledge of the law, and with the expectations of the community. They were responsible for finding the balance between guilt, criminal responsibility, and the impact of crime on victims.

In some interviews, participants described instances when the justice system had worked to further entrench existing disadvantage. Young people in out-of-home-care and Indigenous young people were recognised as especially likely to become caught up in a cycle of systemic failures, while one participant gave an example of a young person who became traumatised in custody due to long periods of isolation and the failure of services to provide adequate mental health care.

*Unfortunately, a child who appears to be ignoring court orders, staying on bail, curfews, not reoffending, they're all things that are very hard for a child with FASD to be able to navigate and if they play up, they're likely to have their bail breached and end up in custody. We know that especially Aboriginal children and Aboriginal and Torres Strait Islander children are exposed to that at a higher rate and behaviour is criminalised. So they're sucked into the vortex of the criminal justice system and it's very difficult for them to escape without the support that they actually need. (Source: Interview #11)*

Participants described the ways in which they adjusted their communication approaches to uphold their clients’ rights. In some instances, this meant altering their language and using visual aids to ensure that the young person understood the proceedings, while for others it meant ensuring that young people provided informed consent for any interventions. Some participants believed that young people had internalised society’s negative expectations of them, seeing themselves as without worth. In order to divert them on to another pathway these participants focused on empowering young people and providing them with hope for the future.

*I think that's the really important thing, that they're not defined as kids who are repeating crimes. … Because their opinion of themselves is so low, and their sense of worth is so - they feel they have experienced so much devaluing, and so much powerlessness that they are not sure that they can be re-integrated into society in a different way, so that they're not identified or seen as criminals. (Source: Interview #22)*

Finally, it was noted that the bureaucratic structures under which some services operated could be difficult to navigate, with young people and their families facing significant barriers to access them. Participants described instances of young people who were unable to access the extra supports they needed to cope in mainstream education; and who faced financial exclusion, for example if they were unable to gain access to disability support through the NDIS.

Most participants, from magistrates to police officers to service providers in the welfare sector, recognised that the justice system does not work in isolation, but is part of a broader social context. Decision-makers needed to weigh up the different roles of the justice system, in order to protect victims of crime, and to divert perpetrators from lives of crime. Staff in other roles focused on the need to advocate for young people who had often faced significant disadvantage throughout their lives.

## Discussion

This research was undertaken to determine the support needs of the justice sector workforce in a regional area of New South Wales. Results from these interviews indicate a sector in the process of change in relation to knowledge, skills and practice regarding FASD and other neurodevelopmental disabilities. The drivers of this change are varied, including personal interest on the part of police officers, staff in the legal system, and other service workers, as well as increased interest at the policy level from both State and Federal governments (Commonwealth of Australia Department of Health, 2018; Elliott, 2015; Reid, 2018). Across the criminal justice system, FASD and neurodevelopmental disabilities have become increasingly acknowledged as an issue; however, the sector is inadequately prepared and under-resourced to provide adequate screening and assessment, therapeutic or rehabilitative support, and diversion opportunities for these individuals (Cox, Clairmont, & Cox, 2008; Douglas, Hammill, Hall, & Russell, 2012; Douglas, Hammill, Russell, & Hall, 2012; Mutch, Jones, Bower, & Watkins, 2016; Passmore et al., 2018; Townsend, Hammill, & White, 2012). In this study participants reported they often worked outside of their areas of expertise in order to address such gaps.

Results from these interviews suggested that several factors including stigma, the perceived benefit of a FASD diagnosis, and access to FASD diagnostic and management services impacted upon the pursual of a diagnosis. Participants reflected on how these factors affected their own practice, and the decisions of youth, families, and carers. In some cases, participants were wary of pursuing diagnosis because they feared the consequences a label of FASD could have for both the young person and their (often vulnerable) families and/or carers. This is a valid concern, given evidence that family disadvantage can lead to a higher likelihood of a child being placed in foster care (Hamilton & Braithwaite, 2014). However, not diagnosing someone with FASD can also lead to stigma, with parents and/or carers blamed for the child’s behaviour and labelled as ‘bad parents’ (Breen & Burns, 2012). For others, the perceived benefit of a diagnosis was not clear, considered highly complex, and challenging to prioritise in the context of crisis and service disengagement.

Participants reflections on the complex needs of youth involved in the justice system demonstrated that FASD was only one factor amongst many that needed consideration when working with justice – involved youth. Participants also reflected on the complexities of their own roles and the need to have knowledge of a wide range of diagnoses, referral pathways, and treatment and support options. Both factors affected service and workforce engagement, with service gaps linked to staff burnout. Recent research in Australia (Mutch et al., 2016; Passmore et al., 2018) has found that individuals working in the criminal justice system want to know more about FASD. Results of this study revealed a sector that wants to better understand the options available to them when working with a young person with FASD, and for neurodevelopmental disabilities generally. Specifically, participants wanted to know where to direct young people for assessment and diagnosis, and, how to access the extra supports that young people might require. This finding must also be interpreted in the context of a system with time-limited funding, a time-poor workforce, and service siloing. Although the context is different, these findings complement those of Hamilton et al. (2019), which revealed that staff wanted to know how FASD was affecting young people, and how they could access the resources and expertise to improve their work practices.

This study demonstrated a workforce acutely aware of the role that the youth justice system plays in providing welfare to young people when other systems have failed (Richards, 2011). Staff described their need to consider the needs of the young person, the needs of the community, and the requirements of their role or organisation, to achieve better outcomes for the young people they work with. They do so with limited resources, and within the constraints of the existing legislation, often going over and above the requirements of their roles to meet the needs of their clients.

However, this research also revealed some confusion in the definition and understanding of FASD. The terminology for neurodevelopmental disabilities like FASD was interchangeable with mental health, in part due to the legal framework within which services were working. The relevant legislation and case law dealing with offenders with neurodevelopmental disabilities, acquired brain injuries, and mental illness employs several inconsistent and outdated definitions, including using the terms ‘cognitive impairment’ and ‘mental illness’ interchangeably, creating unnecessary confusion and complexity (NSW Law Reform Commission, 2012, NSW Law Reform Commission, 2010). This confusion was reflected in the experiences of participants interviewed for this research. Provisions for young people with neurodevelopmental disabilities (including where FASD was diagnosed or suspected) were made under the same legislation as provisions for young people with mental illness, despite the need for different approaches to sentencing, treatment, and ongoing support.

### Limitations of this study

The study began with a desktop scan of services working with young people who come into contact with the youth justice system, with snowballing used to further recruit participants. As such, the staff interviewed may have already had some interest in FASD. The research was undertaken in an urban economic hub which makes comparisons across geographical regions difficult. The Hunter region, including the local government areas of Lake Macquarie and Newcastle, was included in the National Disability Insurance Scheme (NDIS) trial which ran from 2013 to 2016; the NDIS was rolled out nationally in July 2016. Although the area under investigation is a significant economic centre, diagnostic services are limited, and accessing services in Sydney (150 km away) may not be an option for many young people. There were also reports of variation in service delivery and access within the region, with participants describing barriers to access for young people living in rural areas when compared to the urban centre. It could be that rural services have struggled to adapt to changes to service provision, following the introduction of the NDIS (Dintino, Wakely, Wolfgang, Wakely, & Little, 2019). Further research would be necessary to assess the effects of both the NDIS and regional variation in service delivery and access.

The general scope of this research meant that issues pertinent to the overrepresentation of Indigenous Australians in justice settings (Baldry et al., 2016; Weatherburn, 2014; Weatherburn & Holmes, 2017) could not be explored. It would be valuable for future research to explore the nuances of working with Indigenous youth within the youth justice setting in this region.

At the time of the study, the closest youth detention centre, approximately 95 km from the study area, experienced significant unrest that erupted into violence (Nguyen, Hyams, & Kozaki, 2019). Although staff at the detention centre were not interviewed as part of this research, it is possible that this incident affected the results, particularly for those participants working directly in the courts, police, and youth justice.

## Conclusion and recommendations

Participants working in the youth justice sector in the study area recognised a need to improve their understanding of the impacts of living with a neurodevelopmental disability such as FASD. Participants were dedicated and wanted to know how to best help their clients, but they also risked burnout when interventions were perceived as unsuccessful. This study suggested that justice sector staff wanted access to better information and training to facilitate diagnosis and support for young people with FASD and other neurodevelopmental disabilities. To assist this workforce to provide the most appropriate programs and support to youth who have contact with the justice system, clearer pathways linking diagnosis to service access and specific support are necessary. This is important for young people with FASD, as lack of clarity in the relevance of a diagnosis to accessing services and supports may be the most significant barrier to appropriate support. This research also indicates that service providers in the region would benefit from training with a practical focus, including how to support young people to access diagnosis, and how to advocate for support through the NDIS.

To address the complexities described by staff working with young people in youth justice systems, the following recommendations are made for policy and models of care:

- Adequate screening, assessment and diagnosis processes need to be provided and implemented in a systematic way. This should occur early on and involve systems such as education, health and child protection services.
- The support networks of young people (e.g. families, carers, advocates) would greatly benefit from improvements in sharing of information between services and agencies. This will alleviate staff reliance on personal relationships. The implementation of referral pathway frameworks must ensure youth and families are receiving the most appropriate support.
- Staff are undertaking challenging roles with limited resources to support them. Training should highlight pragmatic initiatives to increase staff confidence in working with vulnerable youth who have neurodevelopmental impairments and understand the implications diagnosis has for the provision of support.
- Importantly, evidence-based therapeutic programs that respond to the young person’s neurodevelopmental needs are required in the community.

### Future work

These findings reinforced the need for a model of care tool to assist staff in understanding and accessing pathways for diagnosis and linking young people to the most appropriate services and supports. The interviews described above contributed to the development of a model of care tool including referral pathways for young people with diagnosed or suspected FASD, in the region. Similar activities have been implemented elsewhere (Appleby et al., 2019; Fitzpatrick et al., 2020). It is hoped that these pathways will improve service communication and the provision of appropriate and organised support for vulnerable youth and their families. To understand the effectiveness of such approaches, future investment must consider the extent to which such work: 1) assists staff in each sector to provide appropriate referrals; 2) assists youth, families and carers in their therapeutic management journey; 3) facilitates early intervention and support for individuals with FASD/ other neurodevelopmental disabilities; and 4) is generalisable to other regions.

## Data Availability

The datasets used and/or analysed during the current study are available from the corresponding author on reasonable request.
